# Effect of Aerobic Exercise Training and Deconditioning on Oxidative Capacity and Muscle Mitochondrial Enzyme Machinery in Young and Elderly Individuals

**DOI:** 10.3390/jcm9103113

**Published:** 2020-09-26

**Authors:** Andreas Mæchel Fritzen, Søren Peter Andersen, Khaled Abdul Nasser Qadri, Frank D. Thøgersen, Thomas Krag, Mette C. Ørngreen, John Vissing, Tina D. Jeppesen

**Affiliations:** 1 Department of Neurology, Copenhagen Neuromuscular Center, Rigshospitalet, DK-2100 Copenhagen, Denmark; ggspa@greve-gym.dk (S.P.A.); khaled_qadri@hotmail.com (K.A.N.Q.); frank.thogersen@gmail.com (F.D.T.); thomas.krag@regionh.dk (T.K.); mette.cathrine.oerngreen.01@regionh.dk (M.C.Ø.); john.vissing@regionh.dk (J.V.); tina@dysgaard.dk (T.D.J.); 2 Molecular Physiology Group, Department of Nutrition, Exercise, and Sports, Faculty of Science, University of Copenhagen, DK-2100 Copenhagen, Denmark

**Keywords:** aerobic exercise training, mitochondria, sarcopenia, endurance, deconditioning, skeletal muscle, elderly

## Abstract

Mitochondrial dysfunction is thought to be involved in age-related loss of muscle mass and function (sarcopenia). Since the degree of physical activity is vital for skeletal muscle mitochondrial function and content, the aim of this study was to investigate the effect of 6 weeks of aerobic exercise training and 8 weeks of deconditioning on functional parameters of aerobic capacity and markers of muscle mitochondrial function in elderly compared to young individuals. In 11 healthy, elderly (80 ± 4 years old) and 10 healthy, young (24 ± 3 years old) volunteers, aerobic training improved maximal oxygen consumption rate by 13%, maximal workload by 34%, endurance capacity by 2.4-fold and exercise economy by 12% in the elderly to the same extent as in young individuals. This evidence was accompanied by a similar training-induced increase in muscle citrate synthase (CS) (31%) and mitochondrial complex I–IV activities (51–163%) in elderly and young individuals. After 8 weeks of deconditioning, endurance capacity (−20%), and enzyme activity of CS (−18%) and complex I (−40%), III (−25%), and IV (−26%) decreased in the elderly to a larger extent than in young individuals. In conclusion, we found that elderly have a physiological normal ability to improve aerobic capacity and mitochondrial function with aerobic training compared to young individuals, but had a faster decline in endurance performance and muscle mitochondrial enzyme activity after deconditioning, suggesting an age-related issue in maintaining oxidative metabolism.

## 1. Introduction

Age-related loss of muscle mass and function, referred to as sarcopenia, is an inevitable process, affecting more than 40% of individuals above 80 years of age [1]. Sarcopenia and reduced aerobic capacity in elderly individuals are strong mediators of morbidity [2] and mortality [3,4]. The ability to perform activities of daily living in healthy individuals is progressively reduced with age, seemingly associated with a decrease in aerobic capacity [5]. Lower levels of aerobic capacity can contribute to a loss of independence, increased incidence of disability, frailty, and reduced quality of life in older people. Sarcopenia and age-related impaired aerobic capacity are related to a multitude of factors, including muscle mitochondrial degeneration [6,7].

It is well established that aerobic exercise training increases maximal aerobic exercise capacity (VO_2peak_), accompanied by improvements in mitochondrial content, function, and enzyme expression in young, untrained individuals [8,9,10,11]. In elderly, findings have been equivocal. Some studies found that 6–16 weeks of intense aerobic exercise training improved aerobic capacity and mitochondrial enzyme activity [12,13,14,15,16,17], while others were not able to confirm significant effects of training in elderly [18,19,20]. Thus, it is unclear whether elderly have an attenuated response to training in aerobic capacity compared with young individuals [13,21,22]; in particular, it is not fully understood whether the plasticity for mitochondrial adaptations to aerobic training occurs to the same extent in young and elderly individuals.

A training-induced increase in muscle mitochondrial content and enzyme activity were shown to return to baseline with as little as 4–8 weeks of deconditioning in healthy, young individuals [23,24,25,26,27]. Thus, aerobic training and deconditioning are effective ways to provoke mitochondrial plasticity. In elderly, it could be hypothesized that the age-associated impairments in aerobic capacity and muscle mitochondrial function could relate to relatively faster loss of mitochondrial capacity with deconditiong, but the effect of deconditioning on mitochondrial content and enzyme activity has never been studied in elderly.

Mitochondria are important for many vital functions of the cell, including being a key initiator of programmed cell death (apoptosis). Studies in rats showed an increased apoptotic activity in the aging muscle, accompanied by a lowered expression of the mitochondrial outer membrane antiapoptotic B-cell lymphoma 2 (Bcl2) protein, which was reversed by 12 weeks of aerobic training [28,29,30]. Furthermore, cleavage of cysteine-dependent, aspartate-specific protease-3 (caspase-3), indicative of increased activation of caspase-3, is a key factor in induction of apoptosis, and it was found to be increased in skeletal muscle of 24-month-old compared to 12-month-old rats [31]. Collectively, these findings in rodents led to the idea that increased apoptotic activity driven by mitochondria could be a contributing mediator of age-related muscle loss in humans that can be reversed by exercise training [32]. These data imply that mitochondria-driven apoptosis could be a key factor behind age-related muscle function and mass loss. However, studies investigating mitochondrial and apoptotic biomarkers in skeletal muscle of elderly in response to aerobic training and deconditioning—and, thus, potential explanation for age-related muscle mass—are scarce.

The aim of this study was to investigate the effect of aerobic training and deconditioning on aerobic capacity and muscle mitochondrial function in elderly (>75 years old) and young healthy individuals (age < 30 years old). We hypothesized that elderly individuals would have indices of mitochondrial dysfunction, but that elderly would increase their aerobic and endurance capacity, as well as measures of mitochondrial content and function, to the same extent as the young individuals after aerobic training.

## 2. Materials and Methods

### 2.1. Individuals

The aim was to include a minimum of 10 elderly healthy individuals (age above 75 years old) and 10 healthy young individuals at the age of 20 to 30 years old. Exclusion criteria were nonsedentary, illness that required medication other than antihypertensive and antithrombotic treatment, severe musculoskeletal pain, neurological disorder, smoking, cardiovascular disease, attendance rate below 80% of total training sessions, additional training during the training phase, or failure to comply with instructions of inactivity during the deconditioning phase. Sedentary was defined as performing less than one hour of exercise a week at low to moderate intensity or a maximum of 5 km of cycling for transportation a day.

All participants completed a detailed medical history and electrocardiography, and all had a normal neurological examination before entering the study.

In total, 21 healthy individuals, 11 elderly (four women and seven men; 80 ± 4 years) and 10 young (five women and five men; 24 ± 3 years) individuals were included in the study. Every included participant completed the study in full.

All individuals gave oral and written consent to participate according to the Helsinki declaration. The study was approved by the Ethics Committee of the Capital Region (No. KF-293615). The individuals were all informed about the nature and risks of the study and gave written consent to participate before inclusion.

### 2.2. Study Design

**Figure 1:**
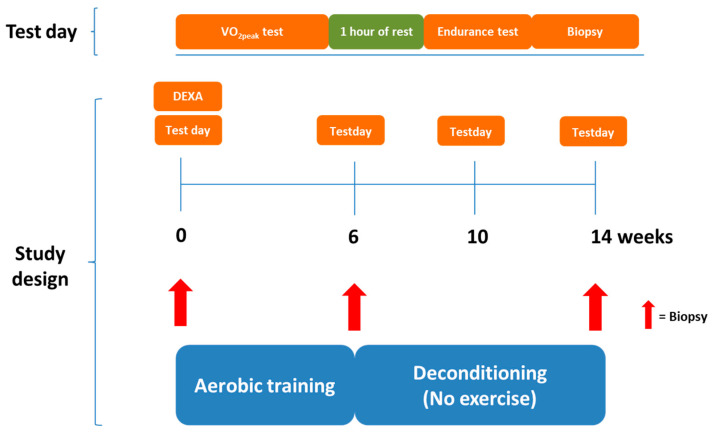
Study design overview. Twenty-one participants completed a 6 week aerobic exercise training intervention followed by 8 weeks of deconditioning (detraining—no exercise). Maximal aerobic exercise capacity and aerobic endurance capacity were evaluated using a maximal oxygen consumption rate test and an endurance time-to-exhaustion test, respectively, before and after aerobic exercise training and after subsequent 4 and 8 weeks of deconditioning. Skeletal muscle biopsies were taken from vastus lateralis muscle at baseline, after 6 weeks of aerobic training and after 8 weeks of deconditioning. DEXA: Dual-energy X-ray absorptiometry, VO_2peak_: Peak oxygen consumption rate.

The 11 elderly and 10 young participants completed a 6 week aerobic exercise training intervention on a bicycle ergometer followed by 8 weeks of deconditioning (Figure 1). Maximal aerobic exercise capacity (VO_2peak_) and maximal workload were evaluated by an incremental test and aerobic endurance capacity evaluated by a time-to-exhaustion test at 80% of pretraining maximal workload before and after aerobic exercise training, and again after 4 and 8 weeks of deconditioning. Skeletal muscle biopsies were taken from vastus lateralis muscle before and after aerobic exercise training and after 8 weeks of deconditioning for measurement of mitochondrial and apoptotic markers. Dual-energy X-ray absorptiometry (DEXA) scanning of body composition was performed at baseline.

### 2.3. DEXA Scanning

A whole-body dual-energy X-ray absorptiometry (DEXA) scan (GE Medical Systems, Lunar, Prodigy, Chicago, IL) was performed prior to the intervention. Elderly and young individuals were instructed to drink 2.5 L of liquid and, hence, be well hydrated the day before the DEXA scan. They arrived overnight-fasted and were encouraged to empty their bladder prior to the scan. They underwent the DEXA scan by lying straight and centered on the table with the hip region within two sets of hash marks on either side of the long edge of the table to ensure the entire body was within the scan area according to the manufacturer’s instructions. The images were analyzed using enCORE™2004 Software (v.8.5) (GE Medical Systems, Lunar, Prodigy, Chicago, IL, USA). Reliability of this DEXA scanning procedure was recently described [33].

### 2.4. Maximal Oxygen Consumption Test

Before initial testing, individuals were familiarized with the equipment and test protocol on a separate day with a training session to reduce the impact that skill learning has on strength performance.

On each test day, individuals carried out an incremental cycling test to exhaustion on a stationary bicycle (Monark 939E, Sweden), and VO_2_ was measured by pulmonary gas exchange with a breath-by-breath gas analyzer using an open-circuit online respirometer for indirect calorimetry measurements (Cosmed, Quark B2, Pavona, Italy). Load was set individually, increasing every other minute for the first 10 min, and thereafter every minute until exhaustion. Heart rate (HR) was measured during exercise, and the subject’s self-assessed feeling of exertion, on a Borg scale, was assessed every minute. Maximal workload (Wmax) was the maximal power output (in Watt) achieved and sustained for at least 1 min during the incremental test.

### 2.5. Endurance Test

After the incremental test, individuals rested for 1 h before carrying out an endurance test on a stationary bicycle (Monark 939E, Sweden) evaluating time to exhaustion at 80% of pretraining Wmax, obtained under the test for maximal oxygen consumption. Exhaustion was achieved when individuals could not maintain a self-chosen pedal cadence rpm minus 10 rpm for 10 s (e.g., if a chosen rpm at 70 dropped to less than 60 rpm for more than 10 s, exhaustion was achieved). During this test, VO_2_ was measured by pulmonary gas exchange as described above, and HR was also measured continuously throughout the test. Exercise economy during the endurance test was calculated as average VO_2_ during the test divided by the workload (Watt).

### 2.6. Aerobic Exercise Training and Deconditioning Interventions

During the 6 week aerobic exercise training intervention, volunteers trained four times per week on a cycle ergometer. Each session lasted 35 min, and sessions alternated between continuous exercise bouts and intermittent exercise bouts. Continuous exercise sessions involved 35 min of continuous cycling at an intensity of 70% of the maximal heart rate (HRmax) reserve (the dynamic area between the resting HR (HRrest) and HRmax). Intermittent exercise consisted of 5 × 4 min intervals at an intensity of 95% of HRmax reserve, with 3 min of rest between intervals.

HRmax reserve has been shown to be well correlated to the intensity as percentage of VO_2peak_. Heart rate intervals were estimated using the following formula, described by Swain et al. (2000) [34]:

HR% intensity = (HRmax − HRrest) × Intensity (%) + HRrest.

Heart rate intervals were set to the calculated HR ± 5 bpm. Training was carried out in a progressive manner, with an increasing workload during the training period to achieve the determined HR intervals. All training sessions were supervised to ensure correct exercise intensity and were carried out on stationary bikes (Monark 939E, Sweden or Tunturi T6, Finland). Heart rate was recorded during exercise by a heart-rate monitor. After 6 weeks of aerobic training, participants stopped the training program and returned to their habitual sedentary lifestyle and were instructed not to initiate any new form of training for the following eight weeks. Individuals wore a step counter during the entire study, i.e., the training and deconditioning period, to ensure that the level of daily activity during the training period corresponded to the activity level of the deconditioning period. Step counters were checked once per week throughout the period to ensure that the physical activity level did not vary more than 10% on a weekly basis.

### 2.7. Skeletal Muscle Biopsies

A skeletal muscle biopsy was performed after the endurance test in vastus lateralis right leg muscle pre- and post-training and after 8 weeks of deconditioning within 15 min of the endurance test. The biopsy was performed as previously described using a 5 mm percutaneous Bergström needle [35]. Needle entry was at least 3 cm away from the previous insertion to avoid scar tissue and interference with data due to post-biopsy edema. Muscle samples were immediately frozen in liquid isopentane cooled by liquid nitrogen before storage at −80 °C for later analysis.

### 2.8. Mitochondrial Enzyme Activities

Citrate synthase (CS) and mitochondrial complex I–IV enzyme activities were determined as previously described [23,36]. Muscle tissue was homogenized in 19 volumes of ice-cold medium containing protease and phosphatase inhibitor cocktail. Enzyme assays for CS and complex I–IV were performed at 25 °C in a Lambda 16 spectrophotometer (Perkin Elmer) [37]. Complex I specific activity was measured by following the decrease in absorbance due to the oxidation of nicotinamide adenine dinucleotide (NADH) at 340 nm with 425 nm as the reference wavelength. Sample was added to a buffer containing 25 mM potassium phosphate (pH 7.2), 5 mM MgCl2, 2 mM KCN, 2.5 mg/mL antimycin A, 0.13 mM NADH, 0.1 mg/mL sonicated phospholipids, and 75 μM decylubiquinone. Complex I activity was measured 3–5 min before addition of 2 μg/mL rotenone, after which the activity was measured for an additional 3 min. Complex I activity was the rotenone-sensitive activity [37,38]. Complex II specific activity was measured by following the reduction of 2,6-dichlorophenolindophenol (DCPIP) at 600 nm. Samples were preincubated in buffer containing 25 mM potassium phosphate (pH 7.2), 5 mM MgCl_2_, and 20 mM succinate at 30 °C for 10 min. Antimycin A (2 μg/mL), 2 μg/mL rotenone, 2mM KCN, and 50 μM DCPIP were added, and a baseline rate was recorded for 3 min. The reaction was started with decylubiquinone (50 μM), and the enzyme-catalyzed reduction of DCPIP was measured for 3–5 min [37,38].

Complex III specific activity was determined in a reaction mixture containing the sample and 100 μM ethylenediaminetetraacetic acid (EDTA), 0.2% defatted bovine serum albumin (*w/v*), 3 mM/L sodium azide, and 60 μM/L ferricytochrome c in 50 mM/L potassium buffer (pH 8.0). The reaction was started by addition of 150 μM decylubiquinol in ethanol and monitored for 2 min at 550 nm [39]. Complex IV activity was measured by following the oxidation of cytochrome c (II) at 550 nm with 580 nm as the reference wavelength. The reaction buffer contained 20 mM potassium phosphate (pH 7.0) and 15 μM cytochrome c (II). Sample was added to the reaction buffer, and the initial activity was calculated from the apparent first-order rate constant after fully oxidizing cytochrome c [37,38].

CS activity was measured following the NADH changes at 340 nm at 25 °C by 50-fold dilution in a solution containing 100 μM acetoacetyl-CoA, 0.5 mM NAD (free acid), 1 mM sodium malate, 8 μg/mL malate dehydrogenase, 2.5 mM EDTA, and 10 mM Tris-HCl (pH 8.0). Samples were preincubated with 0.25% Triton X-100.

### 2.9. Western Blotting Analysis

Western blot analysis was performed as previously described [23,40]. For Western blotting, biopsies were sectioned on a cryostat (Microm HM550, Thermo Fisher Scientific, Waltham, MA, USA) at −20 °C and homogenized in ice-cold lysis buffer mixed with sample buffer. Proteins were separated on an SDS-PAGE gel, blotted to polyvinylidene difluoride (PVDF) membranes, and incubated in primary and secondary antibodies. Antibodies were directed toward Bcl2 (diluted 1:5000; Cell Signalling Technologies, Beverly, MA, USA) and alpha-tubulin (diluted 1:30.000; Abcam, UK, no 4074), with alpha-tubulin used as a loading control. Secondary goat anti-rabbit and goat anti-mouse antibodies coupled with horseradish peroxidase at a concentration of 1:10,000 were used to detect primary antibodies (DAKO, Glostrup, Denmark). Immunoreactive bands were detected by chemiluminescence using Clarity Max, (BioRad), quantified using a GBox XT16 darkroom, and GeneTools software was used to measure the intensities of immunoreactive bands (Syngene, Cambridge, UK). Immunoreactive band intensities were normalized to the intensity of the alpha-tubulin bands for each participant to correct for differences in total muscle protein loaded on the gel.

### 2.10. Bioplex Analysis

Muscle tissue was homogenized in the same way as described above (see Western blotting analysis). The prepared homogenates were diluted to a final protein concentration of 400 μg/mL. The Human Apoptosis 3-plex Panel (Invitrogen, CA, USA) was used for protein quantification of cleaved caspase-3 (cl. caspase-3) and a single-plex magnetic bead assay for beta-tubulin (loading control) (Millipore, Merck KGaA, Darmstadt, Germany). Then, 100 μL of prepared standards were added to separate wells and incubated at room temperature in the dark for 2 h. The plate was washed twice, before adding a 1× detection antibody to the wells, and then incubated for 1 h in darkness at room temperature. The plate was again washed twice, and 50 μL of streptavidin-R-Phycoerythrin (RPE) was added to the wells, followed by 30 min of incubation. The plate was washed three times, and 130 μL of wash solution was added to each well, upon reading the plate on a Luminex Bio-plex 200 system (Biorad, Hercules, CA, USA).

### 2.11. Statistical Analysis

All statistical analyses were carried out using SigmaPlot 11.0 and GraphPad PRISM 8 (GraphPad, La Jolla, CA, USA). All data are expressed as the mean ± standard error of mean (SE), except for baseline anthropometric characterization of participants shown as mean ± standard deviation (SD) (Table 1). A Shapiro–Wilk test was performed to test for normal distribution of data. The differences among groups were analyzed by a repeated-measures two-way analysis of variance (ANOVA) followed by Tukey’s multiple comparison tests, when ANOVA revealed significant interactions. Baseline subject characteristics were evaluated with unpaired *t*-tests between young and elderly groups. Correlation analyses were performed with the Pearson’s product-moment correlation coefficient. Differences were considered statistically significant when *p* < 0.05.

## 3. Results

### 3.1. Anthropometry

Height, total body weight, and fat-free mass were similar among the young and the elderly individuals, whereas the elderly had a higher BMI (+15%), fat mass (+40%), and body fat% (+31%) and a lower VO_2peak_ (−40%) compared with the young individuals (*p* < 0.05; Table 1).

### 3.2. Functional Parameters of Aerobic Capacity

**Figure 2:**
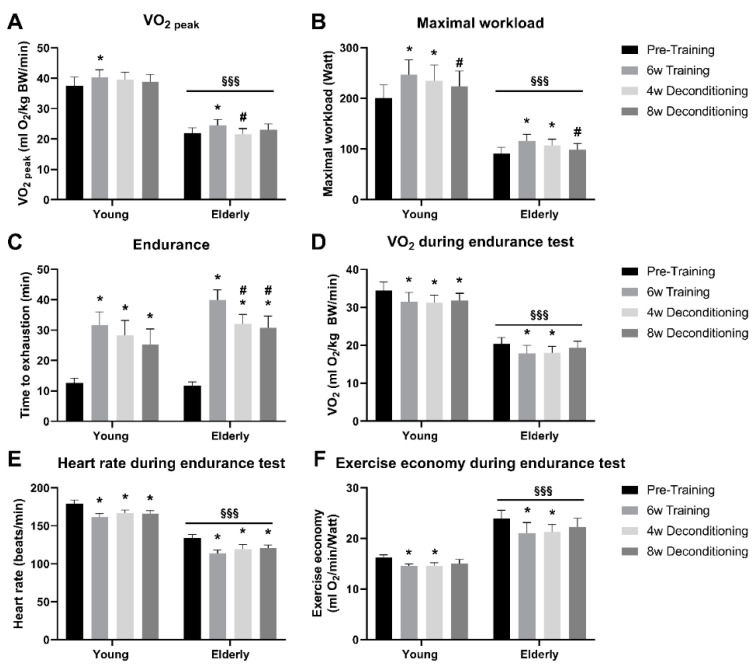
Functional parameters of aerobic capacity. (**A**) Maximal oxygen consumption rate (VO_2peak_) and maximal workload (**B**) measured in an incremental bicycle test before and after 6 weeks of aerobic exercise training and after 4 and 8 weeks of subsequent deconditioning in elderly and young men and women. Time to exhaustion (**C**), average VO_2_ (**D**), average heart rate (**E**), and exercise economy (**F**) during an endurance test on bicycle at 80% of maximal workload in elderly and young men and women. *n* = 10 in young and *n* = 11 elderly. * Significantly different (*p* < 0.05) from pretraining within age group. # Significantly different (*p* < 0.05) from 6 weeks of training within age group. §§§ Significantly different (*p* < 0.001) from young participants. All data are presented as means ± standard error (SE).

The elderly individuals had lower absolute values of VO_2peak_ (~40%) and Wmax (~60%) compared with the young individuals (*p* < 0.001; Figure 2A,B). Six weeks of aerobic training improved VO_2peak_ and Wmax by 13% and 34%, respectively, in elderly individuals (*p* < 0.05) and to the same extent by 9% and 26%, respectively, in young individuals (*p* < 0.05) (Figure 2A,B). VO_2peak_ was lowered by 13% already after 4 weeks of deconditioning in the elderly only (*p* < 0.05), and VO_2peak_ returned to baseline in both the elderly and the young individuals after 8 weeks of deconditioning (Figure 2A). In the elderly individuals, Wmax also returned to pretraining level after 8 weeks of deconditioning, while Wmax was still increased by 11% in the young individuals compared with pretraining level (*p* < 0.05; Figure 2B). Endurance capacity, measured as time to exhaustion on 80% of pretraining Wmax, was improved to a similar extent by 2.4- and 1.5-fold in elderly and young individuals (*p* < 0.05), respectively (Figure 2C). Endurance capacity was impaired by 20% and 25% in the elderly by 4 and 8 weeks of deconditioning, but remained at post-training levels in the young individuals during deconditioning (Figure 2C).

VO_2_ (Figure 2D) and heart rate (Figure 2E) during the endurance test were overall ~30% lower in the elderly compared with the young individuals (*p* < 0.001). VO_2_ (Figure 2D) and heart rate (Figure 2E) were ~15% decreased during the endurance test in both the elderly and the young subjects (*p* < 0.05) and remained so during 4 and 8 weeks of deconditioning. Exercise economy during the endurance test was improved by 12% and 10% in elderly and young individuals (*p* < 0.05), respectively, and remained improved after 4 weeks of deconditioning, but returned to pretraining levels after 8 weeks of deconditioning in both groups (Figure 2F). As a consequence of the relatively lower workload compared with oxygen use during the endurance test in the elderly, exercise economy during the endurance test was overall ~30% lower in the elderly compared with the young individuals (*p* < 0.001) (Figure 2F).

### 3.3. Mitochondrial Enzyme Activities

**Figure 3:**
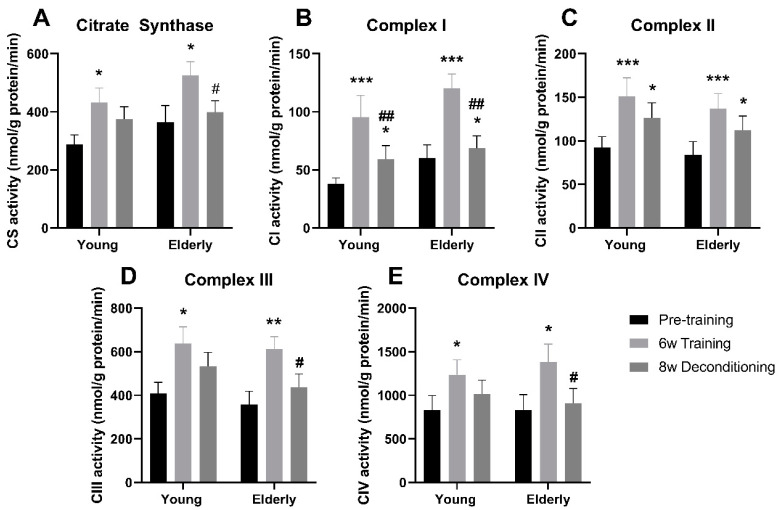
Mitochondrial enzyme activities. Maximal enzyme activity of citrate synthase (CS; **A**), and mitochondrial complex I (**B**), II (**C**), III (**D**), and IV (**E**) in skeletal muscle pre and post six weeks of aerobic exercise training and after subsequent 8 weeks of deconditioning in young and elderly individuals. *n* = 10 in young group and *n* = 11 in the elderly group. */**/*** *p* < 0.05/0.01/0.001, significantly different from pretraining within age group. #,## significantly different from 6 weeks of training within age group.

At baseline, maximal muscle CS and mitochondrial electron transport chain complex I–IV activities did not differ between elderly and young individuals (Figure 3A–E). Six weeks of aerobic training increased muscle CS activity by 31% in elderly individuals (*p* < 0.05), which was the same as that observed in the young individuals (45%) (Figure 3A). Eight weeks of deconditioning decreased CS activity in the elderly individuals by 18% (*p* < 0.05), while CS activity remained at post-training level in young individuals (Figure 3A). The training-induced increases in complex I (163% and 152%; Figure 3B), II (63% and 58%; Figure 3C), III (63% and 49%; Figure 3D), and IV (51% and 40%; Figure 3E) activities were similar in elderly and young individuals. In elderly individuals, 8 weeks of deconditioning decreased complex I (Figure 3B), II (Figure 3C), III (Figure 3D), and IV activities (Figure 3E) by 40%, 8%, 25%, and 26% (*p* < 0.05), respectively, whereas only complex I (26%; Figure 3B) and II (9%; Figure 3C) activities decreased in young individuals after 8 weeks of deconditioning (*p* < 0.05). Interestingly, the change in enzyme activity with deconditioning significantly correlated with change in endurance capacity for complex I (*r* = 0.47, *p* < 0.05) and tended to correlate for complex III (*r* = 0.43, *p* = 0.05) and CS (*r* = 0.39, *p* = 0.09), whereas the change in enzymatic activity for complex II and IV was not significantly correlated to the change in endurance capacity with deconditioning.

When correcting mitochondrial electron transport chain complex I–IV activities individually to CS activity, to take mitochondrial content into account, complex II (−26%) and III (−31%) activities were overall lower in the elderly compared with the young individuals pretraining and also after training and deconditioning (*p* < 0.05), whereas the CS-corrected complex I and IV activity was similar between young and elderly at all time points.

### 3.4. Apoptosis Markers: Cleaved Caspase 3 and Bcl2

**Figure 4:**
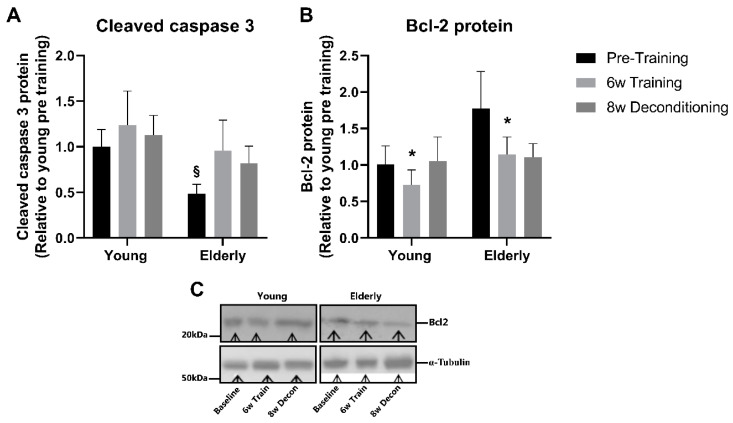
Apoptosis markers. Protein expression of cleaved caspase-3 (**A**) and B-cell lymphoma 2 (Bcl2) (**B**) in skeletal muscle pre and post 6 weeks of aerobic exercise training and after subsequent 8 weeks deconditioning period in young and elderly individuals. *n* = 10 in young group and *n* = 11 in the elderly group; however, due to lack of samples, only *n* = 6 in both groups in (**B**). (**C**) representative Western blots. Values are arbitrary units (means ± SE) and expressed relative to young group pretraining. § *p* < 0.05, young vs. elderly group within pretraining. * *p* < 0.01, main effect of training compared to pretraining independently of age.

There was no effect of aerobic training on cleaved caspase-3 protein content in either the elderly or the young individuals. Pretraining, elderly individuals had a 48% lower expression of cleaved caspase-3 protein content compared with young individuals (*p* < 0.05); however, after aerobic training and deconditioning, there was no longer any difference between the two groups (Figure 4A). Bcl2 protein expression decreased by 21% and 20% after aerobic training to a similar extent in the elderly and young individuals (*p* < 0.05), respectively; however, after 8 weeks of deconditioning, this was not different from pretraining levels in both groups (Figure 4B).

## 4. Discussion

Age-related loss of muscle mass and function may be related to changes in mitochondrial function with age and an impaired response to adapt to the physical activity level. However, only a few studies investigated age-related changes in mitochondrial function in response to aerobic training and deconditioning. In the present study, we investigated age-related changes of aerobic capacity, mitochondrial function, and apoptotic signaling markers with aerobic training and deconditioning and found that (1) only 6 weeks of aerobic training efficiently improved maximal aerobic capacity and mitochondrial function in the elderly individuals, (2) the training effect on aerobic capacity, endurance, mitochondrial enzyme activities, and apoptosis signaling markers in the elderly individuals was similar to that found in the young individuals despite an age difference of more than 50 years, and (3) with deconditioning, the training-induced increases in endurance and mitochondrial enzyme activities decreased in a faster manner in elderly compared with young individuals.

It was suggested that differences in aerobic capacity in elderly versus young healthy humans, at least in part, may be a result of differences in the ability to gain and maintain VO_2peak_ with age [13,21,22]. However, in the present study, 6 weeks of intensive aerobic exercise training resulted in the same increase in VO_2peak_ in elderly individuals compared with that found in young, gender-matched, sedentary individuals, indicating a similar ability to increase oxidative capacity in elderly vs. young individuals. Although the training period was longer than in the present (8–16 weeks), the effect on aerobic capacity was overall the same as previously observed [12,13,14,15,16,17]. The increase in VO_2peak_ in the present study was found after only 6 weeks of aerobic training, suggesting that elderly individuals can increase oxidative capacity after a relatively short training period to the same extent as that seen in young healthy individuals.

Citrate synthase has been shown to be a strong marker of mitochondrial content. Thus, maximal CS activity strongly correlated with mitochondrial volume measured by electron microscopy in skeletal muscles of healthy, young men [41]. Moreover, maximal CS activity correlated with the improvement in mitochondrial volume after 6 weeks of aerobic training in skeletal muscle of young individuals [42]. In the literature, it has been debated whether there is an age-related decline in mitochondrial enzyme activities, since results from studies investigating this have been ambiguous. Several studies found decreased activity of CS and complex I–IV in muscle of elderly [15,43,44,45,46,47], indicating an age-related decline in mitochondrial content and function. Supporting this view, a study investigating the effect of age on mitochondrial content by transmission electron microscopy found a decrease in the content of mitochondria in elderly compared with young individuals [47]. In contrast, in the present study, CS and mitochondrial complex activities were similar at baseline between young and elderly, as we also recently showed between a similar cohort of young (~22 years) and elderly individuals (~82 years) [36] and in accordance with several other studies [13,48,49,50]. Interestingly, when mitochondrial complex activities were corrected relative to CS activity to take mitochondrial content into account, in the present study, complex II and III activities were lower in the elderly compared with the young individuals, implying a loss in the electron transport chain efficiency relative to mitochondrial content. It is possible that the mixed results from studies investigating the effect on age and mitochondrial function in part are related to differences in the pretraining level of physical activity in the investigated elderly individuals. Interestingly, in the present study, elderly individuals were able to increase CS activity (+31%) and mitochondrial complex activities (ranging between 61–163%) with training that matched that found in the healthy young individuals, which emphasizes that the ability to respond to an increase in demand in muscle enzymes in tricarboxylic acid (TCA) cycle and oxidative phosphorylation is preserved at least to the eighth decade of life. The few studies that investigated CS and/or mitochondrial complex activities in elderly of 60–80 years of age did not compare results to young but found a similar increase after 12–16 weeks of training [13,15,16,43], suggesting a similar ability of elderly of 60 and 80 years of age to respond to aerobic training. A recent study with only 6 weeks of high-intensity exercise training also showed improved CS activity and mitochondrial complex protein contents in “younger” elderly (63 years old) men and women [12], which, together with the intense protocol in the present study, underscores that mitochondria can adapt to even short-term training interventions in elderly of both 60 and 80 years of age, when the intensity and frequency of the training are high.

In addition to the exercise training intervention, we included a subsequent deconditioning period to evaluate mitochondrial dynamics in aged human skeletal muscle, which, to our knowledge, has not been studied previously in elderly healthy individuals. Deconditioning after exercise training was investigated in a few studies in healthy young individuals, and data implied that oxidative capacity, muscle mitochondrial protein content, and enzyme activities return to pretraining levels after 6–8 weeks in young individuals [23,24,25,26,27]. In the present study, mitochondrial content judged by CS and mitochondrial complex activities returned to pretraining levels after 8 weeks of deconditioning in the elderly, which was not seen in the young healthy individuals. Thus, enzyme activity of CS and complex I, III, and IV decreased in the elderly to a larger extent than in the young individuals. This indicates that the turnover rate of mitochondrial enzymes in the TCA cycle, as well as the oxidative phosphorylation, is fast and even more rapid in skeletal muscle of elderly. Thus, it seems as the ability to obtain oxidative capacity and increase mitochondrial volume with intensive aerobic training is preserved with age, but is lost faster in aged than in young muscle during subsequent deconditioning. A sedentary lifestyle in elderly individuals may, therefore, be even more deleterious to muscle health than in young individuals.

Endurance capacity is essential in order to maintain independence, reduce incidence of disability, and sustain a high quality of life in older people. In the present study, we found that 6 weeks of intensive aerobic exercise resulted in a remarkable increase in the time to exhaustion during an endurance test in the elderly individuals by 2.4-fold. Moreover, this training-induced increase in endurance was likely, at least in part, mediated by an improved exercise economy, reflecting the capacity to turn oxygen consumption into mechanical work and, hence, lower usage of VO_2_ at the given power. This finding suggests a functional relevance of the training-induced increase in muscle mitochondrial respiratory enzyme activities, through an improved ability to sustain a high energy-production and also a more energy-efficient power production over a longer period of time. The present study is, to our knowledge, the first to demonstrate that the effect on aerobic capacity and muscle mitochondrial function and efficiency seemingly translates into functional improvement of endurance and exercise economy in elderly. In this line, it should be mechanistically studied in future investigations whether aerobic exercise training prevents sarcopenia by improving mitochondrial function and dynamics [51]. Interestingly, with deconditioning, a faster decrease in endurance capacity was observed among elderly compared with young individuals in accordance with similar decreases in CS and mitochondrial complex activities, indicating that, although elderly individuals improve endurance with training in the same manner as young individuals, aerobic endurance seems to be lost faster in elderly individuals, likely related to enhanced degradation of muscle mitochondrial enzymes. To support this notion, we found, despite the modest number of participants in the present study, that the loss of enzyme activity of CS and complex I and III in response to deconditioning tended to correlate to the reduction in endurance capacity. Of note, the faster decline in mitochondrial enzyme activity with deconditioning was in the present study observed in elderly of ~80 years of age, and it remains to be clarified whether 60–75-year-old individuals that are often investigated in the scientific literature would be more affected by deconditioning compared with young individuals. Importantly, a faster decline in endurance performance during deconditioning contrasts with the loss of strength performance after 6 weeks of resistance training, which we recently showed to be similar between young and elderly individuals (~82 years) after comparable 8 weeks of deconditioning [36].

Even though some studies in rodents indicated that apoptosis may play a role during muscle senescence [32], the involvement of age-related apoptosis of skeletal muscle and its regulation with training and deconditioning is poorly understood. Caspase-3 plays an important role in mediating cell death, and Bcl2 is thought to be an antiapoptotic driver. Interestingly, we found a lower muscle content of active caspase-3 (cleaved caspase-3) and a similar Bcl2 expression in the elderly compared with young individuals at baseline. This indicates, at least in healthy elderly individuals, that markers of the muscle intrinsic apoptotic pathway are not upregulated. These findings contrast with findings in rodents, in which increased apoptosis in old muscle of rodents was suggested on the basis of findings of an increased expression of proapoptotic marker cleaved caspase-3 protein [31] and a lower expression of the antiapoptotic Bcl2 protein [28,29,30]. Moreover, in the present study, caspase-3 activity remained similar in elderly and young skeletal muscle after 6 weeks of aerobic training, indicating that exercise training does not induce a higher apoptosis activity. In contrast, Bcl2 protein content decreased slightly in response to training to the same extent in young and elderly, implying either less antiapoptotic signaling after training independently of age or that Blc2 content is not directly coupled to apoptosis rate. To our knowledge, this study is the first to investigate apoptotic markers with training and deconditioning in human muscle of elderly compared with young individuals. Although we only investigated a few markers of a complex signaling, the present results do not substantiate the hypothesis that increased apoptosis with time is the mediator of age-related muscle mass. The faster decline with deconditioning in endurance capacity and mitochondrial enzyme activity could relate to an age-related decline in mitochondrial fusion/fission regulation or an impaired matching of lysosomal mitophagy flux to the demand in aged muscle during deconditioning [51]. In support of the latter, we previously showed in young individuals that 3 weeks of one-legged aerobic training improved the capacity for autophagosomal formation [40], which is also found to occur in elderly [52], emphasizing the importance of physical activity to improve or maintain lysosomal mitophagic capacity. From studies in rodents [53,54,55] and humans [52], it is known that both muscle disuse and aging are associated with impaired mitophagy regulation, and it is, hence, likely that impaired mitophagy and mitochondrial function with deconditioning contribute to accelerated impairment in elderly, which should be addressed in future studies. Overall, accelerated decline in mitochondrial function and sarcopenia seems not to be driven by increased muscle apoptosis in human muscle, and further investigations are needed to elucidate the responsible molecular mechanisms driving sarcopenia and age-related inactivity-induced mitochondrial impairments.

The present study had some limitations that must be acknowledged. It was suggested that potential sex-specific adaptations to aerobic training exist [12]. We recognize that the present study included both men and women but that the number of participants was not optimal to detect an intervention × sex interaction; however, the present study was primarily designed to investigate the effects of training and deconditioning in elderly vs. young individuals. Studies with more subjects are warranted to evaluate potential sex-specific age-related adaptations to training and deconditioning.

## 5. Conclusions

In the present study, we found that 6 weeks of aerobic training efficiently improved maximal aerobic capacity and mitochondrial function in elderly individuals to the seemingly same extent as in young individuals despite an age difference of more than 50 years. This implies that aerobic exercise training is a potent tool to combat age-related loss of aerobic capacity and mitochondrial function. However, with deconditioning, we present the novel finding that the training-induced increases in performance and mitochondrial enzyme activities seemingly decreased in a faster manner in elderly compared with young individuals. This accelerated loss of mitochondrial function in the elderly with deconditioning could play a role in the development of mitochondrial dysfunction and sarcopenia during aging, and responsible mechanisms need to be investigated further in future studies.

## Figures and Tables

**Table 1:** General demographic data.

Demographic Parameter	Young Group	Elderly Group
Age, years	24 ± 3	80 ± 4 ***
Height, cm	175 ± 13	169 ± 9
Weight, kg	70 ± 14	76 ± 14
BMI, kg/m^2^	22.5 ± 2.5	26.5 ± 3.5 *
FFM, kg	50.7 ± 14.3	48.4 ± 10.5
FM, kg	15.5 ± 6.0	26.0 ± 6.9 **
Body fat, %	24.2 ± 9.9	34.9 ± 7.7 *
VO_2 peak_, mL O_2_/kg/min	37.5 ± 9.0	22.5 ± 6.1 ***

BMI, body mass index; FFM, fat-free mass; FM, fat mass, VO_2peak_, maximal oxygen consumption rate. Data are shown as means ± SD. *n* = 10 in young and *n* = 11 in elderly. */**/*** Significantly different (*p* < 0.05/0.01/0.001) from young group.
